# Exposomic Biomonitoring of Polyphenols by Non-Targeted Analysis and Suspect Screening

**DOI:** 10.1021/acs.analchem.3c01393

**Published:** 2023-07-06

**Authors:** Ian Oesterle, Manuel Pristner, Sabrina Berger, Mingxun Wang, Vinicius Verri Hernandes, Annette Rompel, Benedikt Warth

**Affiliations:** † Department of Food Chemistry and Toxicology, Faculty of Chemistry, University of Vienna, Vienna 1090, Austria; ‡ Fakultät für Chemie, Institut für Biophysikalische Chemie, Universität Wien, Wien 1090, Austria; § Doctoral School of Chemistry, University of Vienna, Vienna 1090, Austria; ∥ Department of Computer Science, University of California Riverside, Riverside, California 92521, United States; ⊥ Exposome Austria, Research Infrastructure and National EIRENE Hub, Vienna 1090, Austria

## Abstract

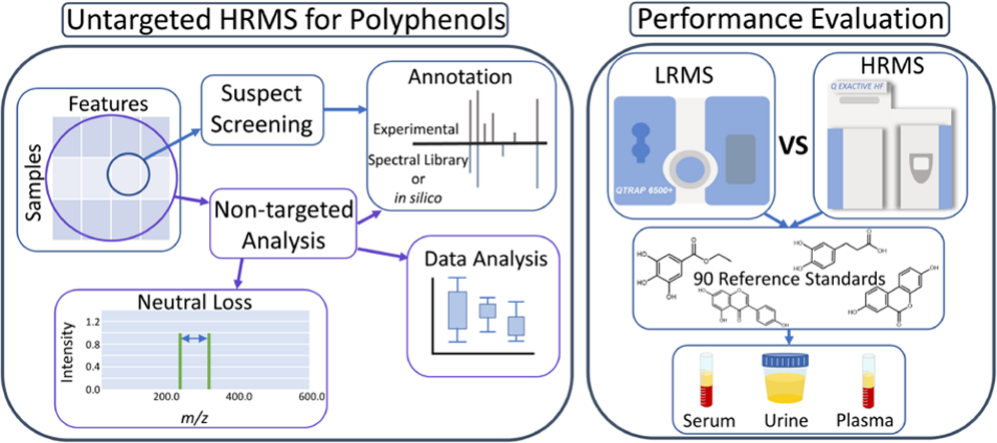

Polyphenols, prevalent in plants and fungi, are investigated intensively in nutritional and clinical settings because of their beneficial bioactive properties. Due to their complexity, analysis with untargeted approaches is favorable, which typically use high-resolution mass spectrometry (HRMS) rather than low-resolution mass spectrometry (LRMS). Here, the advantages of HRMS were evaluated by thoroughly testing untargeted techniques and available online resources. By applying data-dependent acquisition on real-life urine samples, 27 features were annotated with spectral libraries, 88 with *in silico* fragmentation, and 113 by MS^1^ matching with PhytoHub, an online database containing >2000 polyphenols. Moreover, other exogenous and endogenous molecules were screened to measure chemical exposure and potential metabolic effects using the Exposome-Explorer database, further annotating 144 features. Additional polyphenol-related features were explored using various non-targeted analysis techniques including MassQL for glucuronide and sulfate neutral losses, and MetaboAnalyst for statistical analysis. As HRMS typically suffers a sensitivity loss compared to state-of-the-art LRMS used in targeted workflows, the gap between the two instrumental approaches was quantified in three spiked human matrices (urine, serum, plasma) as well as real-life urine samples. Both instruments showed feasible sensitivity, with median limits of detection in the spiked samples being 10–18 ng/mL for HRMS and 4.8–5.8 ng/mL for LRMS. The results demonstrate that, despite its intrinsic limitations, HRMS can readily be used for comprehensively investigating human polyphenol exposure. In the future, this work is expected to allow for linking human health effects with exposure patterns and toxicological mixture effects with other xenobiotics.

A large class of phytochemicals known as polyphenols has been of great interest because they are highly prevalent in plants and fungi and are associated with a variety of beneficial and protective properties in humans, for example, antioxidant^1^ and antibacterial^2^ effects. Moreover, the gut microbiome, known for its impact on numerous health conditions, is affected and modulated by polyphenols.^3^ Polyphenols also hold the potential for negative health impacts, such as exhibiting estrogen-like activity^4^ that might be of relevance in mixture toxicology.^5^ Thus, investigating polyphenols and their metabolites in humans would aid in understanding the correlation between diet and diseases.^2^

As exogenous compounds to the human body, polyphenols are included in the exposome, that is, the total burden of exposure of an individual in a lifetime and its health related effects.^6^ Currently, different exposomic approaches are used^7^ to better understand and quantify the complex exposure of xenobiotics in humans. These approaches often utilize liquid chromatography coupled with mass spectrometry (LC-MS) in two distinct acquisition strategies.^8^ The first, called traditional human biomonitoring or targeted, focuses on acquiring LC-MS data for only specific known analytes of interest using available reference standards.^9^ The second strategy, known as untargeted, involves acquiring spectrometric data on all compounds that can be ionized and are sufficiently abundant.^10^ The untargeted approach can be split into suspect screening, which involves identifying the unknown chemical features by matching different parameters (e.g., monoisotopic mass) with compounds of interest present in databases; and into non-targeted analysis, which includes various techniques such as annotation by *in silico* fragmentation and/or finding relevant features by statistical analysis.^8,9^

Identifying the chemical features in untargeted datasets is a complex endeavor. Since several chemical formulas can have similar monoisotopic masses, especially for larger masses, at least the fragmentation spectra of the features (MS^2^) are needed for tentative compound identification. Data-dependent acquisition (DDA) is one of the main strategies to acquire MS^2^ spectra. In this strategy, either a certain number of the most intense ions from the MS^1^ scan or ions with defined mass-to-charge ratio (*m/z*) are fragmented.^11^

Different types of mass spectrometers are used depending on the analytical strategy and the objectives of a certain study, as there is a tradeoff between sensitivity and resolution of the instruments. Targeted workflows typically use sensitive instruments equipped with low-resolution mass analyzers, for example, triple quadrupole. In contrast, untargeted workflows use instruments with lower sensitivity but high resolution, achieved with mass analyzers such as quadrupole-orbitrap or quadrupole-time-of-flight, leading to increased accuracy of the measured *m/z* values, which is critical in aiding identification. However, with recent developments in mass spectrometry, the sensitivity of HRMS is nearing that of LRMS, notably for low molecular weight compounds such as xenobiotics.^12,13^

Both of these approaches have been applied to investigate polyphenols, however, they were mainly applied on foodstuffs^14^ rather than human matrices,^15^ especially for untargeted workflows.^16^ Thus, the aim of this study was to transfer a previously validated method for a vast set of polyphenol standards^17^ from a targeted LRMS to an untargeted HRMS platform. The HRMS workflow was developed using suspect screening and four different non-targeted analysis approaches on urine samples from a pilot study. This allowed for a thorough test of untargeted data analysis techniques, evaluation of available online resources, and exploration of potential limitations. As HRMS is known to be less sensitive than LRMS, this gap was quantified to better characterize the applicability of HRMS on polyphenol analysis in human biofluids. This was achieved by comparing the limits of detection (LODs) of both workflows for 90 polyphenol reference standards from a variety of classes. Finally, both workflows were applied to case study samples to compare their performance at naturally occurring concentrations.

## Experimental Section

### Reagents, Solvents, and Chemicals

Authentic reference standards, reagents, and solvents were purchased from various sources and are provided in Table S1. The reference compounds were diluted in pure methanol to a concentration of 1 mg/mL, considering the purity and density at 20 °C. The individual reference analyte solutions were diluted and mixed together in methanol at various concentrations to prepare working mixes for spiking.

### Sample Preparation

The urine samples from the case study and the spiked urine, plasma, and serum samples for the LOD comparison were prepared using the optimized protocol by Oesterle *et al.*(17) The samples were stored at −80 °C until analysis. The concentrations of the spiked samples are reported in Table S2. Enzymatic hydrolysis of glucuronides and sulfates was intentionally not performed in this work to be able to perform neutral loss queries of the features.

### LC-HRMS Instrumentation and Parameters

A Vanquish UHPLC (Thermo Fisher) coupled to a QExactive HF quadrupole-Orbitrap (Thermo Fisher) with a heated electrospray ionization source was used for the LC-HRMS measurements. The same LC conditions were used as described in Oesterle *et al.*(17) to best compare the high- and low-resolution measurements, except that eluent B consisted of 0.1% v/v formic acid and 3% v/v water in acetonitrile. Thus, the gradient was adapted and started at 5.2% B, after a 2 min hold, it increased linearly to 66% B within 10 min. B was then set to 97.9% and held for 2 min, before being decreased to 5.2% for a final 2 min hold.

MS^1^ and MS^2^ data of the case study samples were acquired with DDA of the top 10 most intense peaks in negative polarity. The parameters were set with a scan window of 100 to 1100 Da, a full scan resolution of 60,000, MS^2^ resolution of 30,000, a stepped normalized collision energy of 10, 30, and 50 eV, and the additional parameters listed in Table S3. For each biological sample, an iterative exclusion list was prepared with IE-Omics,^18^ until either five injections or the maximum length of the exclusion list (5000 features) was reached.

For the LOD comparison, full scan mode from 62 to 900 Da at a resolution of 120,000 was used. Data was acquired for both positive and negative polarities, separately. Additional MS parameters are listed in Table S3. A total of 10 technical replicates were acquired in each polarity at three concentration levels over the linear range: low, medium, and high, corresponding to levels 3, 5, and 7, respectively in Table S2.

### Data Analysis

The acquired DDA data was first converted from raw files into MGF files using MSConvert (v3.0.22067).^19^ MZMine (v3.1.0)^20^ was used for feature preprocessing and extraction of the MS^2^ data from the MGF files, for MS^1^ screening of the features with the PhytoHub^21^ and Exposome-Explorer^22^ databases, and for spectral library matching with a library from MS-DIAL composed of databases such as MassBank, ReSpect, and GNPS.^23^ To determine molecular formulas and predict structures, *in silico* fragmentation was performed in SIRIUS (v5.5.7)^24^ with CSI/FingerID,^25^ COSMIC,^26^ and ZODIAC.^27^ Features of interest from MS^1^ matching or *in silico* fragmentation were further matched for annotation with METLIN Gen2 spectral library.^28^ All parameters selected for MZMine, METLIN, and SIRIUS are listed in Table S3. The features with MS^2^ spectra were additionally screened for sulfate or glucuronide neutral losses with MassQL^29^ on GNPS, as described in Table S3. The MassQL queries are also listed in Table S3. MetaboAnalyst (v5.0) was used for statistical analysis.^30^ The programming language R (v4.2.0) was used for making plots and performing Van Krevelen analysis.^31^ The NTA Study Reporting Tool (SRT) was used as a guideline for reporting the various components of the developed workflow.^32,33^

The MS^1^ data acquired for the LOD value comparison was analyzed and evaluated with Skyline (v21.2).^34^ The actual concentrations of the analytes were calculated using the area of their chromatographic peaks and a 1/*x* weighted calibration curve. Standard addition was applied and evaluated in Excel if a chromatographic peak was present in the blank samples of the calibration curve. The concentrations of the detected analytes in the case study samples were corrected for extraction efficiency using values previously determined^17^ and dilution factor.

The LOD was calculated by first dividing the standard deviation of the concentration of the 10 spiked technical replicates with the square root of the number of replicates and multiplying this value by 3, as advised in the Eurachem guideline^35^ (Table S3). The LODs were then multiplied by 20, the dilution factor of the sample preparation.

### Biological Samples, Case Study, and Approval to Use Human Matrices

The case study samples were collected as individual 24 h pool from four volunteers at three different time points. The 3 days were: after a polyphenol washout, after consuming a high polyphenol smoothie in the morning, and after a day following a high polyphenol diet, as described in Oesterle *et al.*(17) The sources of pooled human serum and Li-heparin plasma used for the LOD value comparison are listed in Table S1. The urine used for the matrix matched calibration curve and LOD comparison originated from 24 h pooled urine acquired from a female volunteer after following a low polyphenol diet for two days. The University of Vienna ethics committee, under authorization number #00650, approved the collection and measurement of samples from the participants following receiving their signed consent.

## Results and Discussion

### Suspect Screening of Polyphenols and the Exposome in Real-Life Urine Samples

To detect polyphenols and other xenobiotics along with their biotransformation products present in human urine, a suspect screening workflow was developed. However, as urine is an extremely complex and dynamically changing biofluid, these analytes are likely not the most abundant features present. Thus, iterative exclusion DDA was applied to get better coverage of all the features present.^18^ While the focus of this work was on polyphenols, the additional screening for other xenobiotics and endogenous metabolites, should highlight the potential of the HRMS approach for comprehensive exposure and effect analysis.

A total of 6060 features were detected, of which 3742 had MS^2^ data. To assess which features were potentially polyphenols, the 6060 features were matched by their monoisotopic mass with the PhytoHub database, one of the largest online databases on polyphenols containing currently 2268 analytes.^21^ To screen for additional exposome-related features, the features were matched by MS^1^ with the Exposome-Explorer database, which contains currently 1212 biomarkers for environmental exposure.^22^ Features that were MS^1^ matched and had MS^2^ data were then annotated by matching to several open and online spectral libraries, or by *in silico* fragmentation.

**Figure 1:**
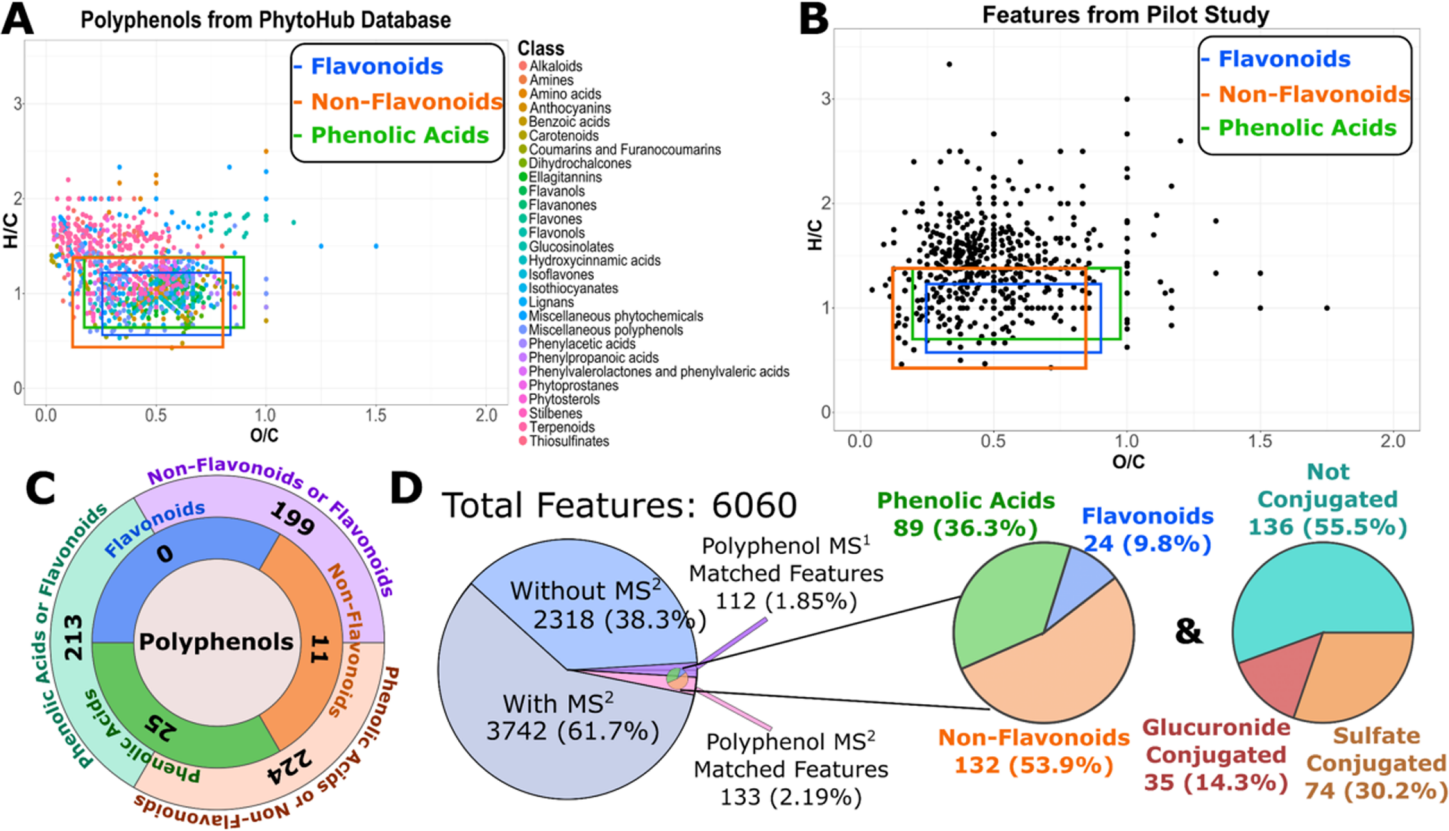
(A) Van Krevelen plot of all >2000 entries in the PhytoHub database. (B) Van Krevelen plot of the features not annotated from the case study samples acquired by DDA which had a molecular formula generated by SIRIUS with a Zodiac score >0.8. (C) A pie chart with the number of unknown features that are potentially polyphenols and classification based on the region they were located on the Van Krevelen plot as shown in (A,B). (D) Pie charts representing the features extracted and the types of polyphenols found from suspect screening.

Feature annotation was based on the identification levels from Schymanski *et al.*(36) but with level 3 split into four sublevels. Features with a library match but for which the correct isomer structure was unknown were labeled as level 3a. Features annotated using *in silico* fragmentation were assigned as level 3b if a tentative structure was known, and level 3c if the elucidated isomer was unknown. Finally, features that were putatively annotated by monoisotopic matching only were assigned a level 3d. For polyphenolic features, a total of 8, 18, 70, and 112 features were assigned to levels 3a–d, respectively. Additionally, 26 features were annotated as level 2a and 11 features could be fully identify at level 1. All of the identified and annotated polyphenolic features are listed in Table S4. From these 245 features, 89 were assigned to phenolic acids, 24 to flavonoids, and 132 to non-flavonoids. These results are displayed in Figure 1d.

When investigating the full range of exposure and effect markers, rather than only polyphenol related features, four additional features were identified as level 1 and 19 annotated as level 2a. Moreover, 6, 23, 13, and 79 additional features were assigned as levels 3a–d, respectively. These features annotated using the Exposome-Explorer database are listed in Table S5.

As a large number of the annotated features were matched with conjugated analytes, the new MassQL algorithm^29^ was used to aid in confirming if the features had a glucuronide (176.0321 Da) or sulfate (79.9573 Da) moiety. From the 245 polyphenolic features, 74 features indicated a sulfate conjugation and 35 a glucuronide conjugate. For the non-polyphenolic features, six indicated glucuronide conjugation and 12 a sulfate conjugate. These results complement a previous study by Jarmusch *et al.*(29) in which seven metabolites were identified with MassQL to have a sulfate moiety.

Suspect screening of polyphenols in human biofluids is typically employed to determine association between polyphenols and the intake of a certain food.^37^ The results here are similar to previous research, with the majority of the polyphenol metabolites found in human urine being phenolic acids, including many that are either glucuronidated or sulfated. These results further emphasize the importance of biotransformation products, notably conjugated metabolites, when investigating human urine and potentially other biofluids,^38^ which many targeted methods still omit.^6^

### Exploring Additional Potential Polyphenol-Related Features through Non-Targeted Analysis

Untargeted data acquisition leads to a considerable number of features, which not only complicates data analysis but many features are a result of noise or of non-pertinent analytes. Therefore, different approaches are used to simplify the data sets and extract unknown features of relevance. Here, four separate approaches were tested and evaluated to extract features that may be polyphenolic but were not able to be annotated previously by *in silico* fragmentation, MS^1^ or MS^2^ matching. These features are thus labeled as level 4 if a molecular formula was predicted and level 5 if nothing else is known about the feature.

The first approach involved plotting the ratio of hydrogens to carbons (H/C) versus the ratio of oxygens to carbons (O/C) in a compound’s molecular formula, also known as a Van Krevelen plot.^31^ As seen in previous research,^39^ polyphenols tend to aggregate in specific regions of the plot. This aggregation was further exemplified when plotting the entries of PhytoHub (Figure 1a). This region can be split into three parts: phenolic acids, flavonoids, or non-flavonoids. The unknown features which had a molecular formula with a Zodiac score of at least 0.8 were then plotted (Figure 1b). Features were labeled as phenolic acids if they had a H/C between 0.7 and 1.4 and an O/C between 0.13 and 0.9. They were labeled as non-flavonoids if H/C was between 0.5 and 1.4 and O/C between 0.06 and 0.7. Finally, they were labelled as flavonoids if H/C was between 0.6 and 1.3, and O/C between 0.25 and 0.8. This resulted in 224 features listed in Table S6 and depicted in Figure 1c. The issue with this approach is that due to the similarity of the formulas for each of these three groups, the three sections overlap, for example, 199 of the features may be a flavonoid or a non-flavonoid due to this overlap. Moreover, as the entire region of flavonoids overlaps with the other classes, no feature could be indicated as purely flavonoid. Other issues with this approach are that other molecules than polyphenols can fall in the same region of the plot, and it is highly dependent on the accuracy of the molecular formula annotation of the features. Therefore, Van Krevelen plots can be used to easily exclude non-relevant features.

**Figure 2:**
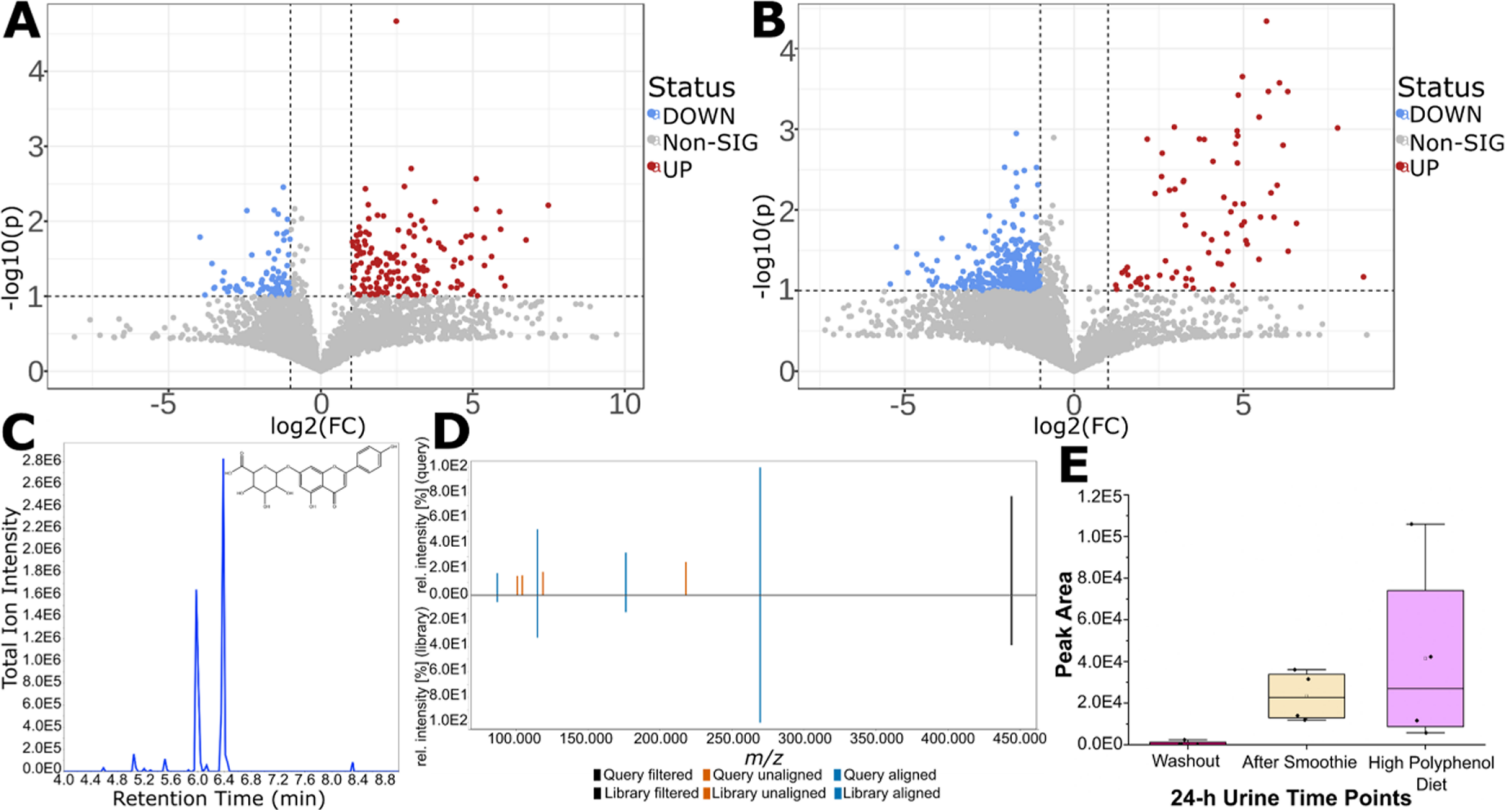
(A) Volcano plot showing the features acquired by iterative DDA of the case study samples which show fold change (FC) versus T-test (*p*), with significance if *p* < 0.1 and FC > 2, between the 24 h urine samples after a washout and after a day of high polyphenol diet. (B) Similar volcano plot as part (A), but between the 24 h urine samples after a washout and after a high polyphenol smoothie. (C) Extracted ion chromatogram from apigenin-7-*O*-glucuronide (*m*/*z* 445.077, RT 6.4), a feature showing significance. (D) Mirror plot of the experimental and spectral library match MS^2^ for the selected feature from (C). (E) Box plot showing the change in peak area between the three different time points for the selected feature from (C).

The second approach involved statistical analysis with MetaboAnalyst,^30^ which allows to easily filter for relevant features. To apply this approach, the pilot study samples were divided into three groups: 24 h urine following washout, 24 h urine following the ingestion of a high polyphenol smoothie, and 24 h urine following a day of a high polyphenol diet. Missing values in the data set were imputed with 1/5 of the minimum positive values. Volcano plots were prepared showing fold change (FC) versus T-test (*p*) between these groups, with significance if FC > 2 and *p* < 0.1 (Figure 2a,b). This allowed the detection of 446 significant features (Table S7). Moreover, as for some features no chromatographic peaks were detected in the washout urine samples, unique features from the other two sample groups were screened for, which yielded 226 features (Table S8). It is important to highlight though that from the 672 features, not all of them are expected to be polyphenolic features. These statistical analysis techniques were also applied to the annotated features (Table S4), which would improve confidence of level 3d matched features as significant features should be polyphenol related. For example, the annotated feature apigenin-7-*O*-glucuronide (level 2a) showed a high fold change of 25 from the washout to the 24 h urine following the consumption of a polyphenol smoothie, and of 44 from the washout to the urine after following a high-polyphenol diet (Table S4 and Figure 2e). Moreover, the extracted ion chromatogram for this feature is shown in Figure 2c, and the MS^2^ spectra with the library match in Figure 2d.

The third approach to filter for polyphenol-related features involved CANOPUS, a useful tool that allows for compound class annotation from the fragmentation spectra.^40^ Features that were labeled with phenol class were filtered, yielding 222 features (Table S9). The advantage of CANOPUS is that structural reference data is not required, but it is prone to errors due to being a computational approximation.

**Figure 3:**
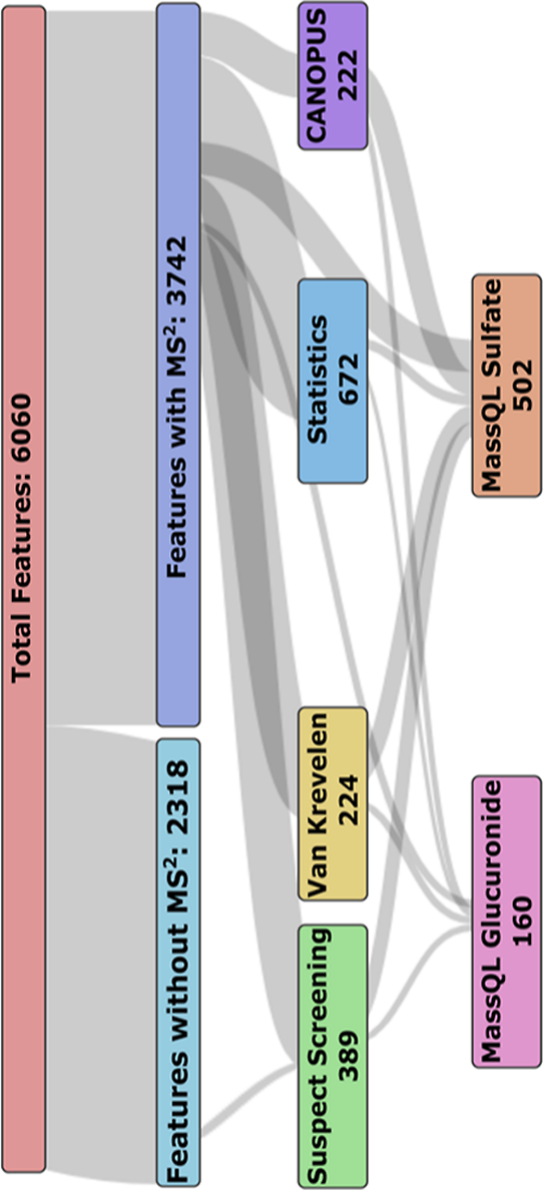
Sankey diagram displaying the number of features extracted from the 24 h urine samples with the various non-targeted analysis techniques and suspect screening.

MassQL can be a powerful tool for polyphenols, as specific fragments can be queried, such as backbones of certain polyphenol classes. Additionally, MassQL can be used for neutral loss queries, such as sulfate and glucuronide loss as done here on the 24 h urine samples. These two biotransformation products were chosen because the suspect screening revealed that a large percentage of the features annotated were conjugated xenobiotics. The two queries yielded 502 features with a sulfate loss (Table S10) and 160 features with a glucuronide loss (Table S11). The relationship between the features detected from MassQL, suspect screening, and the other non-targeted analysis techniques is shown in Figure 3. The same queries were then applied to the raw mzML data to find potentially analytes lost during feature processing, for example, with “non-ideal” peak shapes. These queries yielded an additional 1025 hits (Table S12) for glucuronide loss and 10208 hits (Table S13) for sulfate loss that were not previously determined. It has to be noted that the high number of hits is because these are not features yet but rather refer to an MS^2^ scan. The number of hits were reduced by clustering and grouping the hits by their *m/z* and retention times. Another problem with MassQL are identification errors, for example, false positive results, which depend on the selected parameters, such as the ppm deviation.

### Limitations of Untargeted HRMS

A general obstacle with polyphenols that complicates feature annotation is that they are a chemical class that contains a vast number of positional, constitutional and stereoisomers, which is complexified if human biotransformation products are included. For example, for many features in Table S4 it is difficult to tell which position the conjugation occurs as MS^2^ spectra are extremely similar. Additionally, different unique features were matched with the same analyte, for example, two features with retention times 4.5 and 5.1 min both had a spectral library match with either m-, p-, or o-cresol (Table S4). Chromatographic separation of isomers is a challenging endeavor, as seen here where apigenin and genistein both have the same monoisotopic mass and their retention times are both 8.33 (Table S4). Therefore, they will have the same chromatographic peak and their MS^2^ spectra will be acquired together. This is not an issue in targeted workflows as these two compounds will have distinct quantifier/qualifier ions. This may be overcome in untargeted workflows by adding ion mobility as a complementary dimension to HRMS to separate co-eluting compounds and increase the confidence of structural elucidation.^41^

The main challenge in untargeted approaches is compound annotation and identification. One limitation with this is the size and quality of spectral libraries, especially as different types of mass spectrometers or collision energies yield different MS^2^ spectra. Other techniques can be used for structural annotation of more features, such as *in silico* fragmentation, as seen here with more features annotated at levels 3b and 3c than levels 2a and 3a. However, *in silico* fragmentation has its own pitfalls as it is a computational prediction of MS^2^ spectra. Moreover, *in silico* fragmentation techniques require reference structures as an input and thus would not be suitable for analytes which no structure has been previously described. Being able to elucidate structures from features of completely unknown analytes is extremely difficult, but combining various approaches, such as those described here, can yield valuable insight into the nature of these unknown features. Moreover, the use of complementary approaches can increase the confidence in their results.

Finally, one intrinsic limitation of untargeted workflows is that the HRMS instruments used typically trade-off sensitivity for having higher resolution. Though as not all analytes and instruments perform the same, it is valuable to know the sensitivity of an HRMS workflow to better consider itś applicability in a specific study.

### Comparing the Sensitivity of HRMS and LRMS Platforms Using Spiked Samples

To quantitatively evaluate the difference between the LRMS and HRMS workflows for polyphenols, a sensitivity comparison was performed by calculating the LODs of reference standards (Table S14) in HRMS spiked at a low level (level 2, Table S2) in the three matrices. To keep conditions as similar as possible, the same concentrations were used as in the LRMS method. However, due to the different sensitivities of the instruments, a chromatographic peak could not be integrated for certain analytes. Thus, the LOD calculations were adapted to the number of replicates, or if less than three replicates were available, medium or high spiked samples were used instead.

There are various techniques to calculate LODs. One common technique is to use the signal-to-noise ratio and calculate at which concentration this ratio would equal to 3. However, in both LRMS and HRMS, there is not always a defined background noise level. Therefore, another approach was used which involves calculating the LOD from standard deviation of multiple technical replicates, as recommended in the Eurachem guideline^35^ and used in the LRMS workflow. Though different HRMS acquisition modes are available, such as product ion scan, data were acquired in full scan as it is the most commonly used data acquisition mode and typically one of the first steps in untargeted workflows.

The majority of the 90 model analytes listed in Table S14 were detected in the HRMS measurements with only 16, eight, and nine analytes not found at any spiked concentration in urine, serum, and plasma, respectively (reported as “n.d.” in Table S14). Several analytes had background contamination in the matrix matched “blank”, thus standard addition was applied. The analytes were 3-methylcatechol, 4-hydroxybenzoic acid, enterolactone, and hippuric acid for urine; 3,3-hydroxyphenylpropanoic acid, hippuric acid, and salicylic acid for serum; and 3,3-hydroxyphenylpropanoic acid, benzoic acid, hippuric acid, and salicylic acid for plasma.

**Figure 4:**
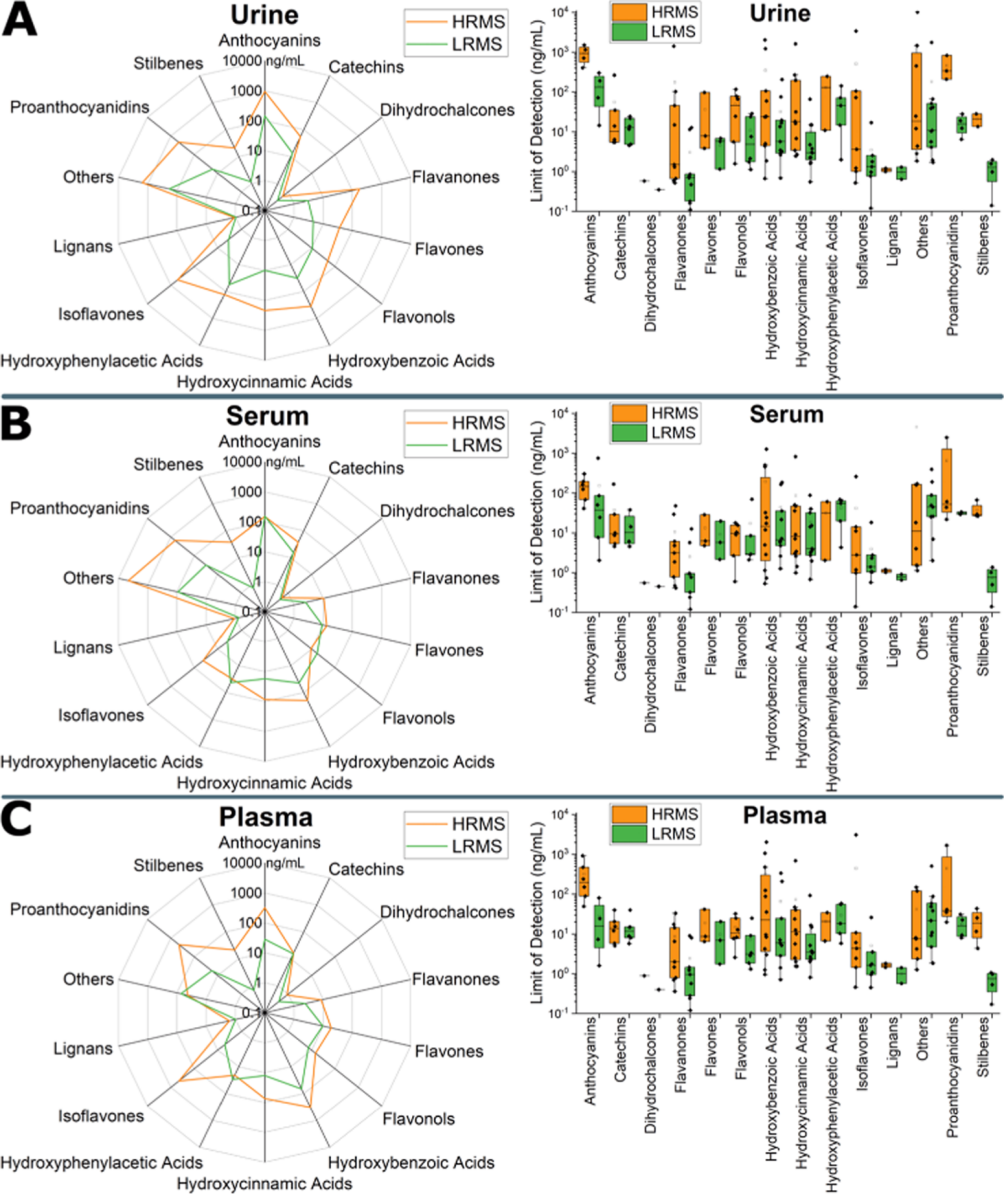
Spider and box plots of the LODs of 90 polyphenols depicting the difference in sensitivity between the HRMS (orange) and LRMS (green) workflows in three different human biofluids: (a) urine, (b) serum, and (c) plasma. LRMS data were reported before by Oesterle *et al.*(17)

Figure 4 and Table S14 illustrate that for urine, 12 out of 90 analytes (13%) displayed a higher sensitivity in HRMS than in LRMS. This value is 29 (32%) for serum and 22 (24%) for plasma. Moreover, the plots in Figure 4 show that the majority of the polyphenol classes have similar average LODs between the two instruments. The median of the LODs of urine, serum, and plasma in the HRMS instrument were 18, 10, and 11 ng/mL, respectively, compared to 4.8, 5.8, and 5.2 ng/mL, respectively, in LRMS. However, the box plots of Figure 4 show that HRMS has a larger variance between the different analytes in each polyphenol class than LRMS.

It is reported in literature that depending on the analytes and instruments chosen, HRMS may perform better or worse than LRMS.^42^ In one study comparing HRMS and LRMS for a multi-class xenobiotic method,^12^ the authors concluded that LRMS would be more suited and the preferred platform for quantification of such compounds. Different from those small molecules, polyphenols are a more homogenous class, present in higher concentrations and with overall better ionization efficiency due to stabilization of proton loss by their aromatic rings and lower background noise in negative ESI mode.^43^ Therefore, as shown here, the sensitivity of HRMS is similar to that of LRMS for many polyphenols, with the additional potential of a broader screening that includes their secondary metabolites (*e.g.,* sulfates and glucuronides).

### Qualitative and Quantitative Comparison in Real-Life Urine Samples

Samples spiked with authentic reference standards are useful when developing workflows. However, comparing the results of HRMS and LRMS platforms in real-life samples yields additional information of the sensitivity gap between LRMS and HRMS.

With the HRMS instrument, 23 out of the 90 selected model polyphenols were detected, while 37 analytes were detected with LRMS.^17^ From these analytes, 18 were detected in both platforms, with their concentrations around a factor 3 lower in HRMS than in LRMS (Table S15). Thus, the 5 analytes that were detected with HRMS but not LRMS could be from the analyte having a better LOD in the HRMS, such as is the case for caffeic acid. Alternatively, it could be a false positive or interference with another analyte of the same MS^1^ mass since in HRMS only a MS^1^ scan was acquired and no fragmentation data.

A difference in determined concentrations between HRMS and LRMS workflows is normal, for example, up to a 20% difference was found for pesticides in various foodstuffs by del Mar Gómez-Ramos *et al.*(44) Though here the difference between the two workflows was a factor of 3, most likely due to human biofluids being more complex matrices than foodstuffs and having more interferences. Although the difference here being larger than in previous research, the majority of the analytes are still detected, showing that HRMS is suitable for the qualitative and semi-quantitative analysis of polyphenols and their metabolites in human samples.

## Conclusion and Outlook

Despite several limitations of untargeted approaches, HRMS can successfully be utilized for investigating the exposome, notably polyphenols and their metabolites in human samples besides many other exogenous and endogenous small molecules. For polyphenols, although HRMS has shown to have lower sensitivity for most of them, the differences are still in the range where HRMS is able to detect—and better characterize—the vast majority of polyphenols. Due to their higher naturally occurring concentration, for example, concentrations ranging from 0.01 μM to over 1000 μM in human urine,^15^ and the more in-depth knowledge about these molecules compared to many other xenobiotics,^12^ HRMS can readily be used instead of LRMS for qualitative and semi-quantitative analysis of polyphenols with a great gain of information. Although targeted LRMS methods are still desired for benchmarking, HRMS has the advantage that it allows to study the exposome agnostically. Currently, polyphenols are frequently under-investigated in human matrices, but workflows such as the one presented here would allow to better investigate both positive and negative effects of these xenobiotics. For example, this workflow can be applied to investigate toxicological and pharmacological mixture effects of polyphenols with other xenobiotics, such as decreasing the potency of potentially harmful xenobiotics or interfering with drug treatment.^45^ Therefore, the authors strongly recommend the use of HRMS in complement to LRMS in the future investigation of exposure and effects of polyphenols and other xenobiotics in human nutritional and health studies.

## Data Availability

The raw data files have been submitted to the Metabolights data repository (MTBLS7564).

## Supporting Information Available

Supporting Information (Excel) is included which contains tables with additional information on the material and methods, and the detailed results from the workflow, such as the LODs for each reference standard or the features filtered by MassQL (XLSX)
Moreover, the completed BP4NTA Study Reporting Tool is included for enhanced reporting confidence (XLSX)
